# SPARC Aggravates Blood-Brain Barrier Disruption via Integrin *α*V*β*3/MAPKs/MMP-9 Signaling Pathway after Subarachnoid Hemorrhage

**DOI:** 10.1155/2021/9739977

**Published:** 2021-11-11

**Authors:** Takeshi Okada, Hidenori Suzuki, Zachary D. Travis, Orhan Altay, Jiping Tang, John H. Zhang

**Affiliations:** ^1^Department of Neurosurgery, Kuwana City Medical Center, 3-11 Kotobuki-cho, Kuwana, Mie 511-0061, Japan; ^2^Department of Neurosurgery, Mie University Graduate School of Medicine, 2-174 Edobashi, Tsu, Mie 514-8507, Japan; ^3^Department of Physiology and Pharmacology, Loma Linda University, Risley Hall, Room 219, 11041 Campus St., Loma Linda, CA 92354, USA; ^4^Department of Earth and Biological Sciences, Loma Linda University, Risley Hall, Room 219, 11041 Campus St., Loma Linda, CA 92354, USA; ^5^Department of Anesthesiology, Loma Linda University, Risley Hall, Room 219, 11041 Campus St., Loma Linda, CA 92354, USA; ^6^Department of Neurosurgery, Loma Linda University, Risley Hall, Room 219, 11041 Campus St., Loma Linda, CA 92354, USA

## Abstract

Blood-brain barrier (BBB) disruption is a common and critical pathology following subarachnoid hemorrhage (SAH). We investigated the BBB disruption property of secreted protein acidic and rich in cysteine (SPARC) after SAH. A total of 197 rats underwent endovascular perforation to induce SAH or sham operation. Small interfering ribonucleic acid (siRNA) for SPARC or scrambled siRNA was administered intracerebroventricularly to rats 48 h before SAH. Anti-SPARC monoclonal antibody (mAb) 236 for functional blocking or normal mouse immunoglobulin G (IgG) was administered intracerebroventricularly 1 h after SAH. Selective integrin *α*V*β*3 inhibitor cyclo(-RGDfK) or phosphate-buffered saline was administered intranasally 1 h before SAH, along with recombinant SPARC treatment. Neurobehavior, SAH severity, brain edema, immunohistochemical staining, and Western blot were evaluated. The expression of SPARC and integrin *α*V*β*3 was upregulated after SAH in the endothelial cells. SPARC siRNA and anti-SPARC mAb 236 prevented neuroimpairments and brain edema through protection of BBB as measured by IgG extravasation 24 and 72 h after SAH. Recombinant SPARC aggravated neuroimpairments and cyclo(-RGDfK) suppressed the harmful neurological effects via inhibition of activated c-Jun N-terminal kinase, p38, and matrix metalloproteinase-9 followed by retention of endothelial junction proteins. SPARC may induce post-SAH BBB disruption via integrin *α*V*β*3 signaling pathway.

## 1. Introduction

Aneurysmal subarachnoid hemorrhage (SAH) is a serious life-threatening cerebrovascular disease arising from a ruptured intracranial aneurysm [1–3]. Following an aneurysm rupture, arterial blood spreads into the subarachnoid space leading to devastating brain injury. This brain injury is caused by blood components, their secondary products, which are induced by tissue damage, and mechanical stress caused by the rapid elevation of intracranial pressure [4–7]. Many secondary products induced by tissue damage have been reported to cause the breakdown of the endothelial junctions forming the blood-brain barrier (BBB), leading to vasogenic edema (which is a major cause of brain injury and an independent risk factor for mortality and poor outcome after SAH [4, 8]). Moreover, enhanced BBB permeability allows immune molecules to migrate into brain parenchyma, which further propagates brain injury [9]. Therefore, BBB disruption is a complication which needs to be addressed in order to improve clinical outcomes in SAH. Indeed, a number of papers suggest that treatment options to preserve endothelial junction proteins to protect BBB could be beneficial in improving neurological functions in experimental SAH models [10]. However, the molecular mechanisms of post-SAH BBB disruption remain unclear.

Secreted protein acidic and rich in cysteine (SPARC), also known as osteonectin and basement-membrane 40, is a type of matricellular proteins (MCPs). SPARC is a secondary product induced in response to injury and exerts diverse functions while the levels of protein expression are low in a steady-state condition, and knockouts of MCPs in mice undergo normal development [11, 12]. SPARC may be involved in BBB disruption in the acute phase of SAH although it has never been investigated as to post-SAH brain injury including BBB disruption. The SPARC levels are elevated in microvascular blood vessels and astrocytes at sites of central nervous system injury [13–15]. An in vitro study demonstrated that exposure to SPARC increased vascular permeability and decreases expression of tight junction marker, zonula occludens (ZO)-1, and occludin [13].

Integrins are a superfamily of cell adhesion receptors that recognize mainly ECMs and cell-surface ligands and are composed of *α* and *β* subunits forming 24 known combinations [16]. Of the integrin family, integrin *α*V*β*3 is located in endothelial cells [17] and recognizes SPARC, activating the downstream signaling involving mitogen-activated protein kinases (MAPKs), proinflammatory mediators such as interleukins (ILs), and matrix metalloproteinase (MMP)-9 [16–24], although previous studies have never been investigated as to integrin *α*V*β*3 signaling pathway in SAH. MMP-9 is a proteolytic enzyme to cause BBB disruption in SAH [4]. In addition, activated integrin *α*V*β*3 induces ZO-1 and occludin internalization, disrupts vascular endothelial- (VE-) cadherin localization, and increased expression of MMP-9 [25]. Therefore, post-SAH SPARC induction may disrupt the BBB via MAPKs/MMP-9 signaling pathway. Thus, the aim of this study was to investigate if SPARC exerts BBB disruption in post-SAH and if SPARC signaling involves integrin *α*V*β*3 and the downstream molecule MAPKs.

## 2. Materials and Methods

The Institutional Animal Care and Use Committee (IACUC) of Loma Linda University (LLU) approved the study protocol (No. 8170018), and all experiments were conducted in accordance with the NIH guidelines for the use of Animals in Neuroscience Research as well as the ARRIVE guidelines. All authors have read and approved the submitted manuscript.

### 2.1. Animal Model

Ten-week-old Sprague-Dawley male rats (weight, 300-320 g) were used to produce SAH models by endovascular perforation as previously described [5]. Briefly, each rat was intubated under deep anesthesia and maintained by mechanical ventilation with 3% isoflurane, and a respiration rate of 77/min for the duration of the surgery. After exposing the left common, external, and internal carotid arteries, a sharpened 4-0 monofilament nylon suture was advanced rostrally into the left internal carotid artery from the external carotid artery stump to perforate the bifurcation of the anterior and the middle cerebral arteries. Sham-operated rats underwent identical procedures except that the suture was withdrawn without puncturing an artery. We monitored peak inspiration pressure and respiratory rate by a pressure-controlled ventilator, heart rate, skin pigmentation, and pedal reflex (firm toe pinch) during operation. These vitals were monitored every 5 min to ensure that animals were not in distress and were responding to the anesthesia and procedure accordingly. Body temperature was kept constant 37°C by a heating pad until the animals recovered. All rats were randomly assigned to the following experimental groups as described (Additional file 1: Figure S1). The time points were selected based on the previous studies, which reported that post-SAH BBB disruption peaked within 24 h and was reversed by 48–72 h following experimental SAH [23, 26]. In the present study, the brain water content was measured at 24 and 72 h postsurgery, and Western blot (WB), except experiment 1, and immunohistochemical staining were performed at 24 h postsurgery in all experiments.

### 2.2. Study Protocol

#### 2.2.1. Experiment 1

To determine the time course and cellular localization of endogenous SPARC and integrin *α*V*β*3 expression, 40 rats were randomly divided and assigned to six groups: sham and SAH 3, 6, 12, 24, and 72 h groups. After mortality and SAH severity were evaluated, WB samples from the left hemisphere were analyzed (*n* = 6 per group). Also, double immunohistochemical staining of SPARC and integrin *α*V*β*3 in conjunction with astrocytes and cerebral endothelial cells was performed in the sham and SAH group at 24 h after modeling (*n* = 2 per group).

#### 2.2.2. Experiment 2.1

To evaluate the effects of the knockdown of SPARC on post-SAH BBB disruption at 24 h, 48 rats were randomly divided and assigned to four groups: sham (*n* = 14), SAH (*n* = 6), SAH + scrambled small interfering ribonucleic acid (Scr siRNA; 500 pmol; *n* = 14), and SAH + SPARC siRNA (500 pmol; *n* = 14) groups. Negative control Scr siRNA (SR30004, OriGene Technologies, Rockville, MD) and SPARC siRNA (200133, Thermo Fisher Scientific Inc., Waltham, MA) were administered intracerebroventricularly (i.c.v) 48 h before modeling. Mortality, neurological scores, SAH severity, brain water content (BWC; *n* = 6 per group), immunohistochemical staining (*n* = 4 per group), and WB (*n* = 4 per group) were evaluated at 24 h after modeling.

#### 2.2.3. Experiment 2.2

To evaluate the effects of the knockdown of SPARC on outcomes at 72 h, 18 rats were randomly divided and assigned into three groups: sham, SAH + Scr siRNA, and SAH + SPARC siRNA (500 pmol) groups (*n* = 6 per group). Negative control Scr siRNA and SPARC siRNA were administered i.c.v 48 h before SAH induction. Mortality, neurological scores, SAH severity, and brain water content were evaluated at 72 h after modeling.

#### 2.2.4. Experiment 3

To confirm the involvement of endogenous SPARC on post-SAH BBB disruption, 30 rats were randomly assigned to three groups: sham, SAH + normal mouse immunoglobulin G (IgG; 0.3 *μ*g), SAH + SPARC monoclonal antibody (mAb) 236 (0.3 *μ*g) groups (*n* = 10 per group). Negative control normal mouse IgG (12-371, EMD Millipore Inc., Temecula, CA) and functional blocking antibody SPARC mAb 236 (AB_2617208, Developmental Studies Hybridoma Bank, Iowa city, IA) were administered i.c.v 1 h after SAH induction. After mortality, neurological scores, and SAH severity were evaluated, brain water content (*n* = 6 per group) and immunohistochemical staining (*n* = 4 per group) were assessed 24 h after modeling.

#### 2.2.5. Experiment 4

To investigate the effects of exogenous SPARC and the involvement of integrin *α*V*β*3 on BBB disruption, 30 rats were randomly assigned to five groups: sham + phosphate-buffered saline (PBS), sham + recombinant human SPARC (rSPARC; 0.3 *μ*g), SAH + PBS, SAH + rSPARC, and SAH + rSPARC + cyclo(-RGDfK) (cRGD; 2.0 *μ*g) groups (*n* = 6 per group). A rSPARC (941-SP, R&D Systems Inc., Minneapolis, MN) was administered i.c.v 1 h after modeling, and a selective integrin *α*V*β*3 inhibitor cRGD (S7834, Selleckchem Inc., Houston, TX) was administered intranasally 1 h before SAH induction. After mortality, neurological scores, and SAH severity were evaluated, WB samples from the left hemisphere were analyzed 24 h after modeling.

### 2.3. SAH Severity and Exclusion Criteria

The severity of SAH was blindly assessed for each experimental animal that was sacrificed as previously described [5, 27]. Briefly, the basal cistern was divided into 6 segments, and each segment was allotted a grade from 0 to 3 depending on the amount of SAH. The rats received a total score ranging from 0 to 18 by adding the scores from all 6 segments. In the analysis, mild SAH (SAH grade ≤ 7) rats were excluded, because mild SAH did not induce neurological impairments [5].

### 2.4. Intracerebroventricular Infusion

An i.c.v infusion was performed as previously described [5]. Briefly, rats were placed in a stereotaxic apparatus under 2-2.5% isoflurane anesthesia. The needle of a 10 *μ*l Hamilton syringe (Hamilton Company Inc., Reno, NV) was inserted through a burr hole into the right lateral ventricle using the following coordinates relative to the bregma: 0.9 mm posterior, 1.5 mm lateral, and 3.3 mm below the horizontal plane of the bregma. Drug and siRNA were infused at a rate of 1 *μ*l/min. The needle was removed 5 minutes after i.c.v infusion, and we quickly sealed the skull hole with bone wax, and then, the incision site was sutured. The dosage of siRNA was determined based on our previous in vivo report [5]. Negative control Scr siRNA and SPARC siRNA were prepared at a concentration of 100 pmol/*μ*l in RNase free resuspension buffer. A total volume of 5 *μ*l siRNA was injected 48 h before SAH induction. The SPARC mAb 236 exerts function blocking effects by the action of the rounding (inhibition of spreading) activity of SPARC on endothelial cells [28]. The dosage of SPARC mAb 236 was determined based on a previous in vitro report [28]. In the study, 1 *μ*g/ml SPARC mAb 236 significantly inhibited the adhesion of SPARC to endothelial cells. The dosage of SPARC mAb 236 was determined to achieve the equivalent working concentration of 1 *μ*g/ml in cerebrospinal fluid (CSF) in rats, whose total CSF volume is presumed to be 300 *μ*l [29], and determined to be 0.3 *μ*g. SPARC mAb 236 and normal mouse IgG were prepared at a concentration of 41 *μ*g/ml in PBS, and a total volume of 7.3 *μ*l of SPARC mAb 236 and normal mouse IgG were infused at 1 h after SAH induction. The dosage of rSPARC was determined based on a previous in vitro study [13]. In the study, 1 *μ*g/ml rSPARC significantly reduced the tight junction marker ZO-1 and occludin in cultured human cerebral microvascular endothelial cells. To achieve an equivalent CSF concentration in rats, the dosage of rSPARC was determined to be 0.3 *μ*g.

### 2.5. Intranasal Administration

Intranasal administration was carried out as previously described [5]. Briefly, rats were placed in a supine position under 2% isoflurane anesthesia. A total volume of 2.0 *μ*g cRGD was dissolved in 4 *μ*l PBS, and administered into the left nares 1 h before modeling. The cRGD has a molecular weight of 717.69 and a molecular formula of C_27_H_41_N_9_O_7_, which can cross the BBB [30]. The dosage of cRGD was determined based on a previous in vitro report [31]. In this study, a concentration of 1.33 nmol/l cRGD showed 50% inhibition of signal (IC_50_ value) for the inhibition of the vitronectin binding to integrin *α*V*β*3. A 10-time higher concentration of IC_50_ value for cRGD was tentatively selected in this study. To achieve an equivalent concentration in rats, total body fluid volume is presumed to be 210 ml (70% of body weight) [32], and the dosage of cRGD was determined to be 2.0 *μ*g.

### 2.6. Neurobehavioral Tests

Neurological impairments were blindly evaluated using the modified Garcia's neurological score and beam balance score system as previously described [5]. Briefly, the evaluation consisted of 6 categories (spontaneous activity, spontaneous movement of four limbs, forepaw outstretching, climbing, body proprioception, and response to whisker stimulation) that could be scored 0 to 3 or 1 to 3. A neurological score was determined by adding the 6 scores to get a final score with 3 being the worst and 18, the best (Additional file 1: Figure S2a). The median score of 3 consecutive trials in a 5 min interval was calculated. A beam balance test investigated animal's ability to walk on a narrow wooden beam for 60 seconds, and we blindly marked a grade from 0 to 4 points as follows: 4 points, walking ≥20 cm; 3 points, walking <20 cm; 2 points, walking but falling; 1 point, not walking while remaining on beam; and 0 points, falling without walking (Additional file 1: Figure S2b). The median score of 3 consecutive trials in a 5 min period was calculated.

### 2.7. Brain Water Content

Brain edema was determined using the wet/dry method as previously described [5]. Briefly, after sacrificing rats under deep anesthesia, the brain was quickly removed, separated into 4 segments (left and right cerebral hemispheres, cerebellum, and brain stem), and weighed immediately as wet weight. The brain specimens were dried in an oven at 105°C for 72 h and weighed again as dry weight. The water content of each specimen was calculated according to the following formula: [(wet weight − dry weight)/wet weight] × 100%.

### 2.8. Immunofluorescence Staining

An immunofluorescence staining was performed as previously described [5]. Under deep anesthesia, rats were sacrificed by transcardial perfusion with 100 ml PBS followed by 2 minutes of 10% neutral buffered formalin. Brains were fixed in 10% neutral buffered formalin for 24 h at 4°C followed by 30% sucrose solution for another 72 h. After being frozen at −80°C, the brain was cut into 10 *μ*m thick coronal sections at 1.0 mm posterior to the bregma using a cryostat (LM3050S; Leica Microsystems, Bannockburn, III, Germany). Slides were washed with 0.01 M of PBS three times for 10 minutes then incubated in 0.3% Triton X-100 in 0.01 M of PBS for 30 minutes at room temperature. After being blocked with 10% donkey serum in 0.01 M of PBS for 1 h at room temperature, the sections were incubated at 4°C overnight with the primary antibody as follows: goat antiglial fibrillary acidic protein (GFAP; 1 : 500; ab104224, Abcam Inc., Cambridge, MA), mouse anti-von Willebrand factor (vWF; 1 : 400; sc-365712, Santa Cruz Biotechnology Inc., Dallas, TX), rabbit anti-SPARC (1 : 500; NBP1-80972, Novus Biologicals Inc., Centennial, CO), rabbit anti-integrin *α*V*β*3 (1 : 50; SC56-07, Novus Biologicals Inc., Centennial, CO), rabbit antiphosphorylated c-Jun N-terminal kinase (p-JNK; 1 : 100; ab131499, Abcam Inc., Cambridge, MA), and rabbit antiphosphorylated p38 (p-p38; 1 : 400; 9211, Cell Signaling Technology Inc., Danvers, MA). Then, the sections were washed with 0.01 M of PBS and incubated with the appropriate fluorescence-conjugated secondary antibodies (1 : 200; Jackson ImmunoResearch Inc., West Grove, PA) for 1 h at room temperature. The slides were observed and photographed under a fluorescence microscope (DMi8; Leica Microsystems Inc., Germany; ×20). The densitometric analysis of p-JNK and p-p38 was performed using Image Pro Plus 6.0 software (Media Cybernetics Inc., Rockville, MD) in the 4 continuous pictures of the left (perforation side) temporal cortex based on our previous study [4].

### 2.9. Immunohistochemical Staining of Immunoglobulin G (IgG)

Immunohistochemical staining of IgG was performed to evaluate BBB permeability using a commercially available kit (Vectastain ABC Peroxidase kit; PK-4004, Vector Laboratories, Burlingame, CA). Coronal brain sections were prepared in the same way as immunofluorescence staining. After being washed with 0.01 M of PBS three times for 10 minutes, slides were incubated in 0.3% Triton X-100 in 0.01 M of PBS for 30 minutes at room temperature. After being incubated in 3% hydrogen peroxide (H_2_O_2_) for 20 minutes to quench any endogenous peroxidase activity, the sections were blocked with 10% rabbit normal serum in 0.01 M of PBS for 1 h at room temperature followed by overnight incubation at 4°C with biotinylated rabbit anti-rat polyclonal IgG and then 1 h incubation at room temperature with an avidin-biotin-horseradish peroxidase complex. Color reactions were developed in diaminobenzidine/H_2_O_2_ solution, and the sections were lightly counterstained with hematoxylin. To evaluate the amount of IgG extravasation, 4 continuous pictures of the left temporal cortex were photographed under light microscope (×20), and the relative quantity of extravasated IgG was calculated by Image Pro Plus 6.0 software (Media Cybernetics Inc., Rockville, MD). The left (perforation side) temporal cortex was selected based on our previous study [4].

### 2.10. Western Blot

The left cerebral hemisphere was separated and utilized. Equal amounts of protein samples (30 *μ*g) were loaded on SDS-PAGE gels, electrophoresed, and transferred onto a polyvinylidene difluoride membrane. The membranes were blocked with 5% bovine serum albumin in 0.01 M of PBS for 2 h at room temperature followed by incubation overnight at 4°C with the following antibodies: rabbit anti-SPARC (1 : 2000; NBP1-80972, Novus Biologicals Inc., Centennial, CO), rabbit anti-integrin *α*V*β*3 (1 : 1000; SC56-07, Novus Biologicals Inc., Centennial, CO), mouse anti-*β*-actin (1 : 5000; sc-47778, Santa Cruz Biotechnology Inc., Dallas, TX), rabbit anti-p-JNK (1 : 1000; ab131499, Abcam Inc., Cambridge, UK), mouse anti-JNK (1 : 5000; 66210-1-Ig, Proteintech Group Inc., Rosemont, IL), rabbit anti-p-p38 (1 : 1000; 9211, Cell Signaling Technology Inc., Danvers, MA), rabbit anti-p38 (1 : 1000; 9212, Cell Signaling Technology Inc., Danvers, MA), rabbit anti-IL-6 (1 : 1000; PR627, Thermo Fisher Scientific Inc., Waltham, MA), rabbit anti-MMP-9 (1 : 1000; ab182734, Abcam Inc., Cambridge, UK), rabbit anti-ZO-1 (1 : 1000; 61-7300, Thermo Fisher Scientific Inc., Waltham, MA), and rabbit anti-VE-cadherin antibody (1 : 1000; ab33168, Abcam Inc., Cambridge, UK). On the following day, the membranes were incubated with the appropriate secondary antibody (1 : 5000; sc-516102, Santa Cruz Biotechnology Inc., Dallas, TX, and 1 : 5000; 12-348, MilliPore sigma Inc., Temecura, CA) at room temperature for 2 h. Immunoreactive bands were detected with a chemiluminescence reagent kit (ECL Prime; Amersham Biosciences Inc., Arlington Heights, IL) and quantified by densitometry with Image J software (NIH, Bethesda, MD).

### 2.11. Statistical Analysis

Neurological score and SAH grade were expressed as median ± 25th–75th percentiles and were compared with Kruskal-Wallis tests followed by Steel-Dwass multiple comparisons. Brain water content, immunohistochemical staining of IgG, and WB results were expressed as mean ± standard deviation and were compared with one-way analysis of variance (ANOVA) followed by Tukey-Kramer post hoc tests. Statistical analyses were performed using SPSS version 23.0 (IBM, Tokyo, Japan). A value of *P* < 0.05 was considered significant.

## 3. Results

### 3.1. General Observation

Comparisons of physiological parameters revealed no significant differences among the groups (data not shown). A total of 197 rats underwent endovascular perforation to induce SAH or sham operation. The mortality was 0 of 54 (0%) in the sham-operated rats, and it was 16 of 147 (11%) in the SAH-operated rats in total during the observation period in all experiments. A total of 15 rats were not used for the analyses because of mild (≤7) SAH grade (Additional file 1: Figure S3a). There was no significant difference in SAH grade among all SAH groups at 24 h after SAH (Additional file 1: Figure S3b, c). Brain water content, immunostaining of IgG, and WB results were normally distributed and had homogeneity of variance.

### 3.2. Time Course and Spatial Expression of SPARC and Integrin *α*V*β*3 after SAH

**Figure 1:**
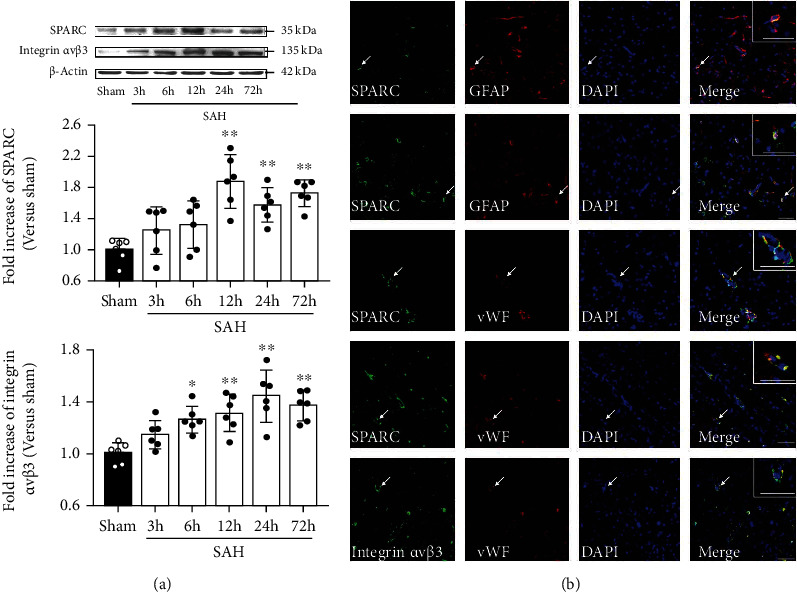
Expression of endogenous secreted protein acidic and rich in cysteine (SPARC) and integrin *α*V*β*3. (a) Representative Western blot images and densitometric quantification of time-dependent expression of SPARC and integrin *α*V*β*3 after subarachnoid hemorrhage (SAH). Expression levels of each protein are divided by the levels of *β*-actin and expressed as a ratio of the average levels of sham models for normalization (mean ± standard deviation; *n* = 6 per group). ^∗^*P* < 0.05 and ^∗∗^*P* < 0.01 vs. sham group. (b) Colocalization of SPARC (green) and integrin *α*V*β*3 (green) with glial fibrillary acidic protein (GFAP; red) or von Willebrand factor (vWF; red) in the left temporal cerebral cortex at 24 h after modeling. Nuclei are stained with DAPI (blue). Inset highlighting higher magnification image of SPARC and integrin *α*V*β*3 colocalized with GFAP or vWF (indicated as arrows). Scale bar = 50 *μ*m (*n* = 2 per group).

WB results showed that expression of SPARC peaked at 12 h after SAH induction, and the elevation was prolonged at least until 72 h when compared to the sham group (Figure 1(a)). In contrast, the expression of integrin *α*V*β*3 was significantly elevated from 6 h to at least 72 h with the peak at 24 h from SAH induction. Double immunofluorescence staining showed that SPARC was expressed in astrocytes and endothelial cells in the cerebral cortex in both sham and SAH groups at 24 h from modeling, and integrin *α*V*β*3 was expressed in endothelial cells at 24 h post-SAH (Figure 1(b)).

### 3.3. Knockdown of SPARC Suppressed Neurological Impairments, Brain Edema, and BBB Permeability after SAH

**Figure 2:**
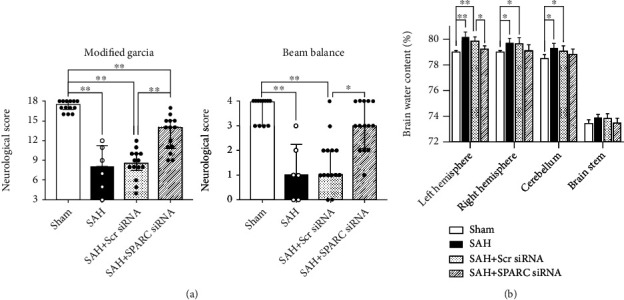
Effects of knockdown of a secreted protein acidic and rich in cysteine (SPARC) on neurobehavioral function and brain edema at 24 h after modeling. (a) SPARC small interfering ribonucleic acid (siRNA) improves modified Garcia's score and beam balance score after subarachnoid hemorrhage (SAH). Data are expressed as median ± 25th‐75th percentiles (*n* = 6‐14 per group). ^∗^*P* < 0.05 and ^∗∗^*P* < 0.01. (b) SPARC siRNA treatment improves brain water content after SAH. Data are expressed as mean ± standard deviation (*n* = 6 per group). ^∗^*P* < 0.05 and ^∗∗^*P* < 0.01. Scr siRNA: scrambled siRNA.

**Figure 3:**
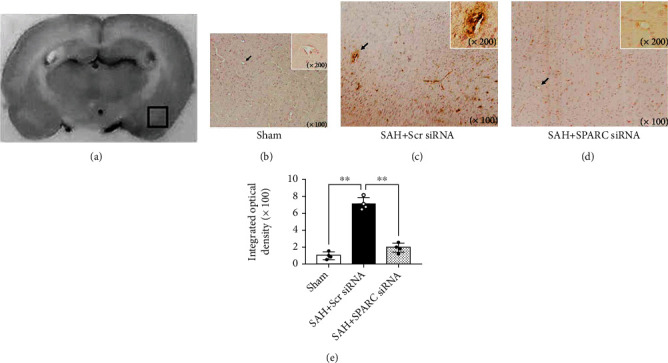
Effects of a secreted protein acidic and rich in cysteine (SPARC) small interfering ribonucleic acid (siRNA) on blood-brain barrier permeability at 24 h after modeling. (a) Representative brain slice showing the location of staining (small box) in the left temporal cortex at 1.0 mm posterior to the bregma. (b–d) Representative immunohistochemical staining of immunoglobulin G (IgG) in sham-operated rats (b), subarachnoid hemorrhage (SAH) rats treated with scrambled siRNA (Scr siRNA) (c), and SAH rats treated with SPARC siRNA (d). Inset highlighting higher magnification image of endothelial cells (indicated as arrows). (e) The sum of integrated optical density of IgG. Data are expressed as mean ± standard deviation (*n* = 4 per group). ^∗∗^*P* < 0.01.

**Figure 4:**
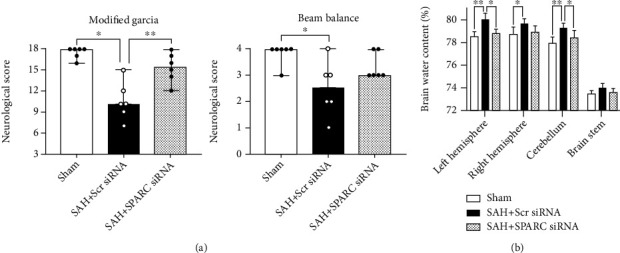
Effects of knockdown of a secreted protein acidic and rich in cysteine (SPARC) on neurobehavioral function and brain edema at 72 h after modeling. (a) SPARC small interfering ribonucleic acid (siRNA) improves modified Garcia's score after subarachnoid hemorrhage (SAH). Data are expressed as median ± 25th‐75th percentiles (*n* = 6 per group). ^∗^*P* < 0.05 and ^∗∗^*P* < 0.01. (b) SPARC siRNA treatment improves brain water content after SAH. Data are expressed as mean ± standard deviation (*n* = 6 per group). ^∗^*P* < 0.05 and ^∗∗^*P* < 0.01. Scr siRNA: scrambled siRNA.

The silencing efficacy of SPARC siRNA was validated by WB and immunofluorescence staining in the left cerebral hemisphere at 72 h after siRNA injection (Additional file 1: Figure S4a, b). After SPARC siRNA was injected, post-SAH aggravation of neurological scores significantly decreased in both modified Garcia's and beam balance tests, while Scr siRNA did not have an effect after SAH (Figure 2(a)). In addition, brain water content in the left cerebral hemisphere 24 h post-SAH significantly improved in the SAH + SPARC siRNA, while Scr siRNA administration had no significant effects on brain water content after SAH (Figure 2(b)). Immunohistochemical staining of IgG was performed to evaluate the severity of BBB disruption in the left cerebral cortex 24 h after SAH (Figures 3(a)–3(d)). SAH + Scr siRNA group resulted in a significant increase in IgG extravasation 24 h post-SAH, which was significantly suppressed in SAH + SPARC siRNA group (Figure 3(e)). At 72 h, SPARC siRNA also inhibited post-SAH aggravation of neurological score in the modified Garcia's test and brain edema in the left cerebral hemisphere and cerebellum compared with Scr siRNA (Figure 4).

### 3.4. Endogenous SPARC May Aggravate Post-SAH Neurological Deficits and BBB Disruption via MAPKs p-JNK and p-p38

**Figure 5:**
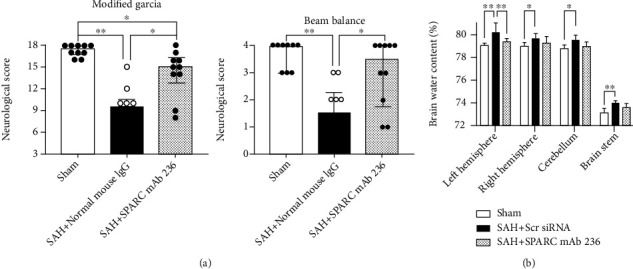
Effects of a functional blocking against secreted protein acidic and rich in cysteine (SPARC) on neurobehavioral function and brain edema at 24 h after modeling. (a) SPARC monoclonal antibody (mAb) 236 improves modified Garcia's score and beam balance score after subarachnoid hemorrhage (SAH). Data are expressed as median ± 25th‐75th percentiles (*n* = 10 per group). ^∗^*P* < 0.05 and ^∗∗^*P* < 0.01. (b) SPARC mAb 236 treatment improves brain water content after SAH. Data are expressed as mean ± standard deviation (*n* = 10 per group). ^∗^*P* < 0.05 and ^∗∗^*P* < 0.01. IgG: immunoglobulin G.

**Figure 6:**
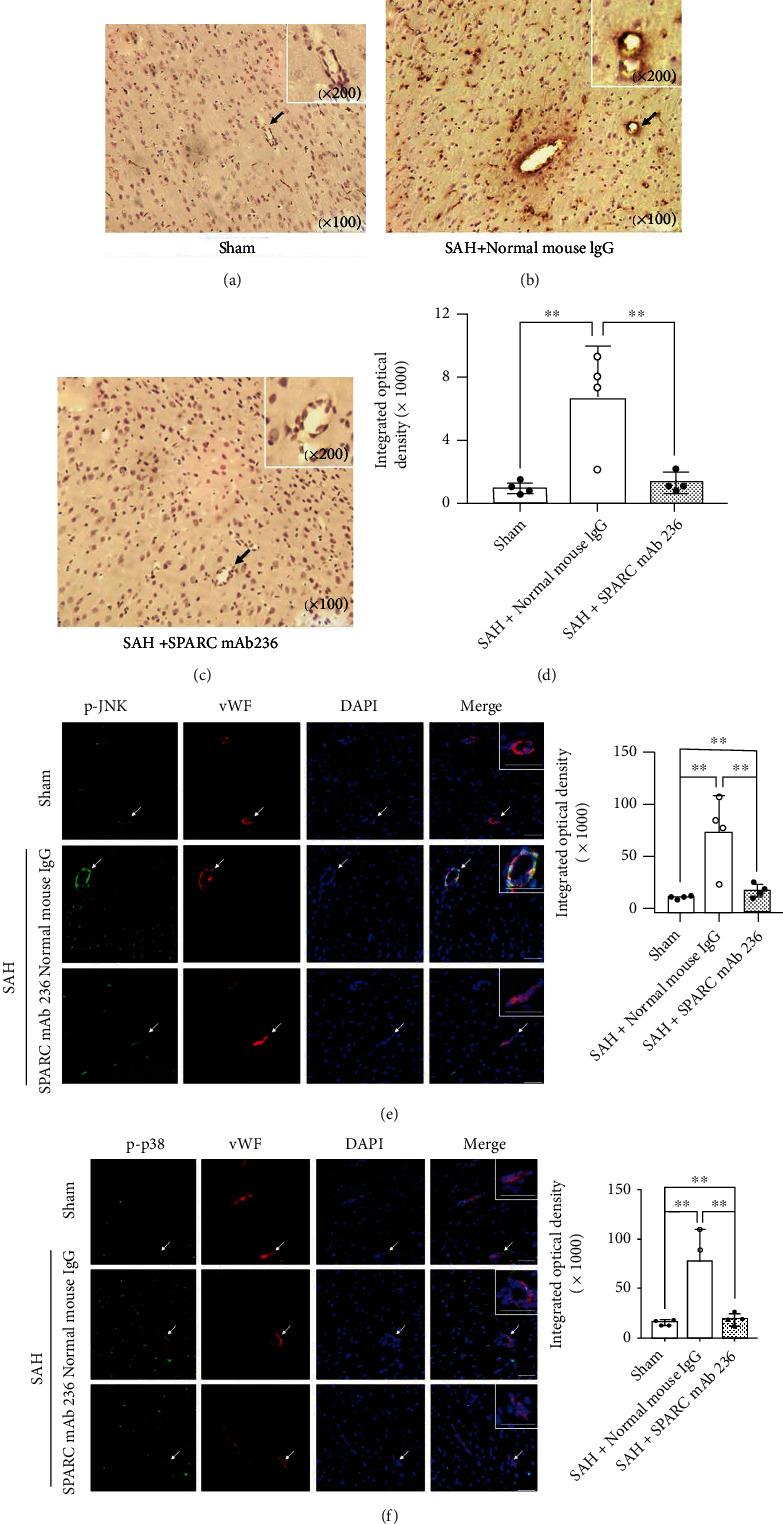
Effects of a functional blocking against secreted protein acidic and rich in cysteine (SPARC) on blood-brain barrier permeability and expression of phosphorylated c-Jun N-terminal kinase (p-JNK) and phosphorylated p38 (p-p38) in endothelial cells at 24 h after modeling. (a–c) Representative immunohistochemical staining of immunoglobulin G (IgG) in the left temporal cortex at 1.0 mm posterior to the bregma in sham-operated rats (a), subarachnoid hemorrhage (SAH) rats treated with normal mouse IgG (b), and SAH rats treated with SPARC monoclonal antibody (mAb) 236 (c). Inset highlighting higher magnification image of endothelial cells (indicated as arrows). (d) The sum of integrated optical density of IgG. Data are expressed as mean ± standard deviation (*n* = 4 per group). ^∗∗^*P* < 0.01. (e, f) Representative immunohistochemical staining of p-JNK (green (e)) and p-p38 (green (f)) in the left temporal cortex at 1.0 mm posterior to the bregma and densitometric analyses of p-JNK-positive cells (e) and p-p38-positive cells (f). Nuclei are stained with DAPI (blue). Inset highlighting higher magnification image of von Willebrand factor (vWF) positive cells (indicated as arrows). Scale bar = 50 *μ*m. Data are expressed as mean ± standard deviation (*n* = 4 per group). ^∗^*P* < 0.05 and ^∗∗^*P* < 0.01.

A SPARC mAb 236 improved neurological scores and brain edema in the left hemisphere compared with negative control normal mouse IgG after SAH (Figures 5(a) and 5(b)). Immunohistochemical staining of IgG showed that SPARC mAb 236 administration resulted in a significant suppression of IgG extravasation 24 h post-SAH compared with normal mouse IgG (Figures 6(a)–6(d)). Expression of two major MAPKs p-JNK and p-p38 in the endothelial cells was assessed by double immunofluorescence staining. SAH rats treated with normal mouse IgG showed the upregulation of p-JNK and p-p38 while SPARC mAb 236 treatment inhibited the expression of p-JNK and p-p38 in endothelial cells. The densitometry of p-JNK-positive cells and p-p38-positive cells were significantly higher after SAH induction, which were significantly suppressed by SPARC mAb 236 administration (Figures 6(e) and 6(f)).

### 3.5. Integrin *α*V*β*3/MAPK Signaling Pathway May Make a Greater Contribution to SPARC-Mediated BBB Disruption after SAH

**Figure 7:**
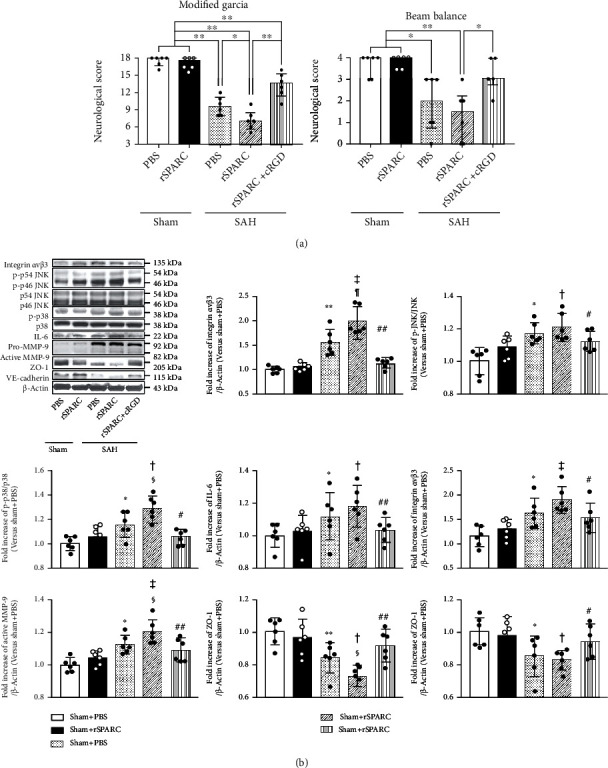
Effects of a recombinant secreted protein acidic and rich in cysteine (rSPARC) and an integrin *α*V*β*3 inhibitor at 24 h after modeling. (a) rSPARC exacerbates modified Garcia's score and inhibition of integrin *α*V*β*3 suppresses the effects of rSPARC after SAH (*n* = 6 per group). ^∗^*P* < 0.05 and ^∗∗^*P* < 0.01. (b) Representative Western blot images and densitometric quantification of expression of phosphorylated p46 and p54 c-Jun N-terminal kinase (p-JNK), phosphorylated p38 (p-p38), interleukin (IL)-6, pro and active matrix metalloproteinase (MMP)-9, ZO-1, and vascular endothelial- (VE-) cadherin. Representative Western blots come from the same samples but multiple membranes. Expression levels of each protein are assessed using *β*-actin as an internal control: the levels are expressed as the target protein/*β*-actin, p-p38/p38, or p-JNK/JNK and as a ratio of the average level of the sham + PBS group for normalization, because the levels of p38 and JNK are unchanged among the groups (mean ± standard deviation; *n* = 6 per group). ^∗^*P* < 0.05 and ^∗∗^*P* < 0.01 vs. sham + phosphate-buffered saline (PBS), ^†^*P* < 0.05 and ^‡^*P* < 0.01 vs. Sham + rSPARC, ^§^*P* < 0.05 and ^¶^*P* < 0.01 vs. SAH + PBS, ^#^*P* < 0.05 and ^##^*P* < 0.01 vs. SAH + rSPARC. cRGD: cyclo(-RGDfK): integrin *α*V*β*3 inhibitor.

A rSPARC did not affect neurological scores in sham-operated rats. However, rSPARC treatment significantly aggravated the neurological scores in post-SAH rats compared with PBS. Selective integrin *α*V*β*3 inhibitor cRGD significantly reduced post-SAH harmful neurological effects of rSPARC (Figure 7(a)). WB analyses also demonstrated that rSPARC did have an effect on the expression of integrin *α*V*β*3 and the intracellular downstream mediators in sham-operated rats. In contrast, post-SAH rSPARC administration enhanced the expression of integrin *α*V*β*3, p-p38, and active MMP-9 and reduced the expression of tight junction marker ZO-1 compared with PBS. The protective effects of cRGD were associated with a significant suppression of post-SAH activation of MAPKs including p-p38 and p-JNK, proinflammatory mediator IL-6, and MMP-9 followed by retention of endothelial tight junction protein ZO-1 and adherens junction protein VE-cadherin (Figure 7(b)).

## 4. Discussion

The novel findings in the present study are as follows: (1) the expression of SPARC and integrin *α*V*β*3 is upregulated in endovascular perforation SAH rats; (2) knockdown of SPARC and functional blocking against endogenous SPARC prevented neurological impairments through protection of the BBB as measured by IgG extravasation after SAH; (3) exogenous SPARC aggravated neurobehavioral impairments after SAH, and integrin *α*V*β*3 inhibitor suppressed the harmful neurological effects of SPARC via inhibition of two MAPKs JNK and p38 and MMP-9 followed by retention of ZO-1 and VE-cadherin.

The post-SAH time course of SPARC and integrin *α*V*β*3 levels have never been determined, while expression of integrin *α*V*β*3 in microvessels after experimental ischemic stroke is significantly increased at 2 h poststroke in adult baboons [33]. The present study demonstrated that the expression of both SPARC and integrin *α*V*β*3 was upregulated within 12 h, and, therefore, SPARC and integrin *α*V*β*3 signaling pathways are possibly involved in acute phase brain injury after SAH. An in vitro study demonstrated that proinflammatory cytokine tumor necrosis factor- (TNF-) *α* increased SPARC expression in cerebral endothelium [13]. In contrast, SPARC knockdown reduced expression of TNF-*α* and IL-6 and protected vascular endothelial cells from damage [34]. In addition, SPARC was regulated by MMPs [35], while SPARC has been shown to induce the production and activity of MMPs [36–38]. SPARC interacting with TNF-*α* and MMPs may amplify the expression levels of each other in an acute phase.

An in vitro study demonstrated that SPARC increased transendothelial albumin flux in a dose-dependent manner at concentrations. At a fixed dose, longer exposure times induced albumin flux, which was blocked by anti-SPARC antibodies [39]. Goldblum et al. [39] demonstrated that SPARC promoted barrier dysfunction in endothelial cell monolayers. Tilling et al. [40] demonstrated that SPARC inhibited collagen IV-mediated increase in transendothelial electrical resistance (TEER) of low TEER brain capillary endothelial cells. TEER value is a widely accepted technique to measure the integrity of tight junction including BBB [41]. The findings of these studies indicated that SPARC increased the permeability of endothelium. However, the effects of SPARC in hemorrhagic stroke have been poorly understood. The present study demonstrated that endogenous SPARC was induced in reactive astrocytes and capillary endothelial cells in an acute phase of SAH and that immediate treatment to block endogenous SPARC exerted BBB protection effects in SAH while exogenous SPARC exerted further harmful neurological effects. These results suggest that SPARC aggravates post-SAH BBB disruption in an acute phase.

BBB is mainly composed of a chemical and structural barrier formed by endothelial junction-associated proteins, such as ZO-1 and VE-cadherin, a basal lamina, a pericyte, and an astrocytic perivascular end-feet [18, 42, 43]. Degradation of the junction-associated proteins opens endothelial cell-cell junction and increases BBB permeability [18, 44]. A proteolytic enzyme MMP-9 has been repeatedly reported to cause post-SAH BBB disruption by degrading the cerebral microvessel basal lamina and endothelial tight junction protein ZO-1 and adherens junction protein VE-cadherin [45–50]. Among the MMP family, MMP-2 also exerts proteolytic effects of endothelial cells only within several hours after experimental ischemic stroke, although other studies have shown no obvious increases in MMP-2 expression [45]. However, MMP-2 activity did not change in brain after filament perforation SAH, while MMP-9 activity was upregulated in our previous study using zymography [51]. Therefore, this study focused on the involvement of SPARC-induced MMP-9 induction after SAH. MCPs other than SPARC also have either beneficial or detrimental effects on BBB via MMP-9 in SAH [18, 24, 42, 46, 49, 52]. Our recent studies demonstrated that three kinds of MCPs, tenascin-C, periostin, and galectin-3, were induced and caused BBB disruption after SAH via MAPK-mediated activation of MMP-9 and subsequent ZO-1 degradation in brain capillary endothelial cells in mice [18, 42, 46, 52]. In contrast, another MCP osteopontin was induced in reactive astrocytes and capillary endothelial cells and prevented MMP-9 activation and BBB disruption by inactivating MAPK and nuclear factor-*κ*B [49, 53]. MAPK activation upregulates proinflammatory cytokines such as TNF-*α*, IL-1*β*, and IL-6, and MMP-9 in SAH [45, 54, 55], and a MAPK inhibitor has been reported to protect the BBB integrity by MMP-9 downregulation and inactivation [56]. The present study showed that exogenous SPARC reduced the expression of tight junction protein ZO-1 via more activation of p38 MAPK and then MMP-9 after SAH. In contrast, integrin *α*V*β*3 inhibitor suppressed the harmful neurological effects of exogenous SPARC via inhibition of two forms of MAPKs JNK and p38 and then MMP-9 followed by retention of ZO-1 and VE-cadherin. Therefore, BBB disruption caused by SPARC induction after SAH possibly depends on integrin *α*V*β*3/MAPK signaling pathway.

There are several limitations in this study that need to be noted. First, the SPARC signaling pathway that would induce post-SAH BBB disruption was not completely teased apart, due in part to the fact that other receptors, MCPs, growth factors, and cytokines have a high affinity with SPARC [21]. Moreover, other downstream mediators besides MAPKs were not examined in this study. A study demonstrated that SPARC has counteradhesive properties that result in rounding of endothelial cells, inhibition of endothelial cell spreading, and loss of focal adhesions through a tyrosine-kinase-dependent pathway [57]. Therefore, other protein-binding SPARC and the downstream signaling pathway influencing post-SAH BBB disruption should be examined in the future. Second, we cannot exclude the possibility that SPARC siRNA and SPARC mAb 236 inactivate other neuroreceptors and neuroregulatory molecules known to be involved in BBB disruption and neurological impairments rather than SPARC. Third, only i.c.v treatment with SPARC siRNA before SAH and SPARC mAb 236 at 1 h after SAH was tested for the effects of SPARC inactivation in this study. As cerebrospinal fluid drainage is often placed in severe SAH patients to promote SAH clearance or to control acute hydrocephalus, an i.c.v. injection of this study could be applied to clinical settings. In order to be more translational, however, the effects of multiple treatments at different dosages or time courses and more simple delivery routes such as intravenous injections should be tested before future clinical trials. Fourth, endogenous ligands that activated integrin *α*V*β*3 other than SPARC were not investigated in this study. For instance, vascular endothelial growth factor, a known inducer of *α*V*β*3 integrin expression, also is upregulated after SAH and causes post-SAH BBB disruption [21, 25, 58]. Further studies are required to determine whether other factors released following SAH stimuli enhance activation of integrin *α*V*β*3. Fifth, we evaluated SAH severity using grading score in addition to neurobehavioral tests. Our previous studies have repeatedly shown that the severity of brain injury was linearly correlated with SAH grading score at least during an acute phase of SAH [5, 59]. However, it would be useful to demonstrate more experimental evidences for successful SAH modeling with uniform brain injuries. In addition, we did not monitor the tissue distribution of SPARC siRNA and mAb and their effects on cerebral blood flow (CBF) and other physiological parameters from postoperation to euthanasia. CBF and physiological parameters affect brain injury during an acute phase of SAH [60]. To the best of our knowledge, although SPARC has not been reported to affect CBF, blood pressure, and other physiological parameters, it would be worth investigating them. Lastly, the verification of the effects on other animal groups with different age, gender, and genetic background is a subject to be addressed in the future [61]. Although additional experiments need to be performed in the future to solve these limitations, the present study is important for providing a novel potential molecular target SPARC against BBB disruption after SAH.

## 5. Conclusion

This study demonstrated for the first time that blockage of endogenous SPARC prevented post-SAH BBB disruption at least via the inhibition of integrin *α*V*β*3/MAPKs/MMP-9 signaling pathway in rats.

## Data Availability

The data used to support the findings of this study are included within the article.

## Supplementary Materials

Supplementary Materials Figure S1: experimental design and animal groups. BWC: brain water content; cRGD: cyclo(-RGDfK): integrin *α*V*β*3 inhibitor; IgG: immunoglobulin G; IHC: immunohistochemistry; mAb: monoclonal antibody; PBS: phosphate-buffered saline; SPARC: secreted protein acidic and rich in cysteine; rSPARC: recombinant SPARC; SAH: subarachnoid hemorrhage; siRNA: small interfering ribonucleic acid; Scr siRNA: scrambled siRNA; WB: Western blot. Figure S2: neurobehavioral tests. (a) Modified Garcia's score. (b) Beam balance test. Figure S3: mortality and subarachnoid hemorrhage (SAH) grade. (a) Animal usage and mortality of all experiment groups. (b) Representative basal image of brain in animal models. (c) SAH grade scores of all groups. Data are expressed as median ± 25th‐75th percentiles. cRGD: cyclo(-RGDfK): integrin *α*V*β*3 inhibitor; IgG: immunoglobulin G; mAb: monoclonal antibody; PBS: phosphate-buffered saline; siRNA: small interfering ribonucleic acid; Scr siRNA: scrambled siRNA; SPARC: secreted protein acidic and rich in cysteine; rSPARC: recombinant SPARC. Figure S4: the knockdown efficiency of a secreted protein acidic and rich in cysteine (SPARC) small interfering ribonucleic acid (siRNA) at 24 h after subarachnoid hemorrhage (SAH) induction. (a) Representative Western blot images and densitometric quantification of expressions of SPARC. Expression levels of SPARC are divided by the levels of *β*-actin and expressed as a ratio of the average levels of sham models for normalization (mean ± standard deviation; *n* = 4 per group). ^∗∗^*P* < 0.01. (b) Representative immunohistochemical staining of SPARC in the left temporal cortex at 1.0 mm posterior to the bregma. Nuclei are stained with DAPI (blue). Scale bar = 50 *μ*m (*n* = 4 per group).
