# Supplementary material for: SPARC Aggravates Blood-Brain Barrier Disruption via Integrin αVβ3/MAPKs/MMP-9 Signaling Pathway after Subarachnoid Hemorrhage
Source: Oxid Med Cell Longev. 2021 Nov 11;2021:9739977. doi: 10.1155/2021/9739977 (PMC8601826; doi:10.1155/2021/9739977)
Supplement: Supplementary Materials — Figure S1: experimental design and animal groups. BWC: brain water content; cRGD: cyclo(-RGDfK): integrin αVβ3 inhibitor; IgG: immunoglobulin G; IHC: immunohistochemistry; mAb: monoclonal antibody; PBS: phosphate-buffered saline; SPARC: secreted protein acidic and rich in cysteine; rSPARC: recombinant SPARC; SAH: subarachnoid hemorrhage; siRNA: small interfering ribonucleic acid; Scr siRNA: scrambled siRNA; WB: Western blot. Figure S2: neurobehavioral tests. (a) Modified Garcia's score. (b) Beam balance test. Figure S3: mortality and subarachnoid hemorrhage (SAH) grade. (a) Animal usage and mortality of all experiment groups. (b) Representative basal image of brain in animal models. (c) SAH grade scores of all groups. Data are expressed as median ± 25th‐75th percentiles. cRGD: cyclo(-RGDfK): integrin αVβ3 inhibitor; IgG: immunoglobulin G; mAb: monoclonal antibody; PBS: phosphate-buffered saline; siRNA: small interfering ribonucleic acid; Scr siRNA: scrambled siRNA; SPARC: secreted protein acidic and rich in cysteine; rSPARC: recombinant SPARC. Figure S4: the knockdown efficiency of a secreted protein acidic and rich in cysteine (SPARC) small interfering ribonucleic acid (siRNA) at 24 h after subarachnoid hemorrhage (SAH) induction. (a) Representative Western blot images and densitometric quantification of expressions of SPARC. Expression levels of SPARC are divided by the levels of β-actin and expressed as a ratio of the average levels of sham models for normalization (mean ± standard deviation; n = 4 per group). ∗∗P < 0.01. (b) Representative immunohistochemical staining of SPARC in the left temporal cortex at 1.0 mm posterior to the bregma. Nuclei are stained with DAPI (blue). Scale bar = 50 μm (n = 4 per group). [file 9739977.f1.docx]

**
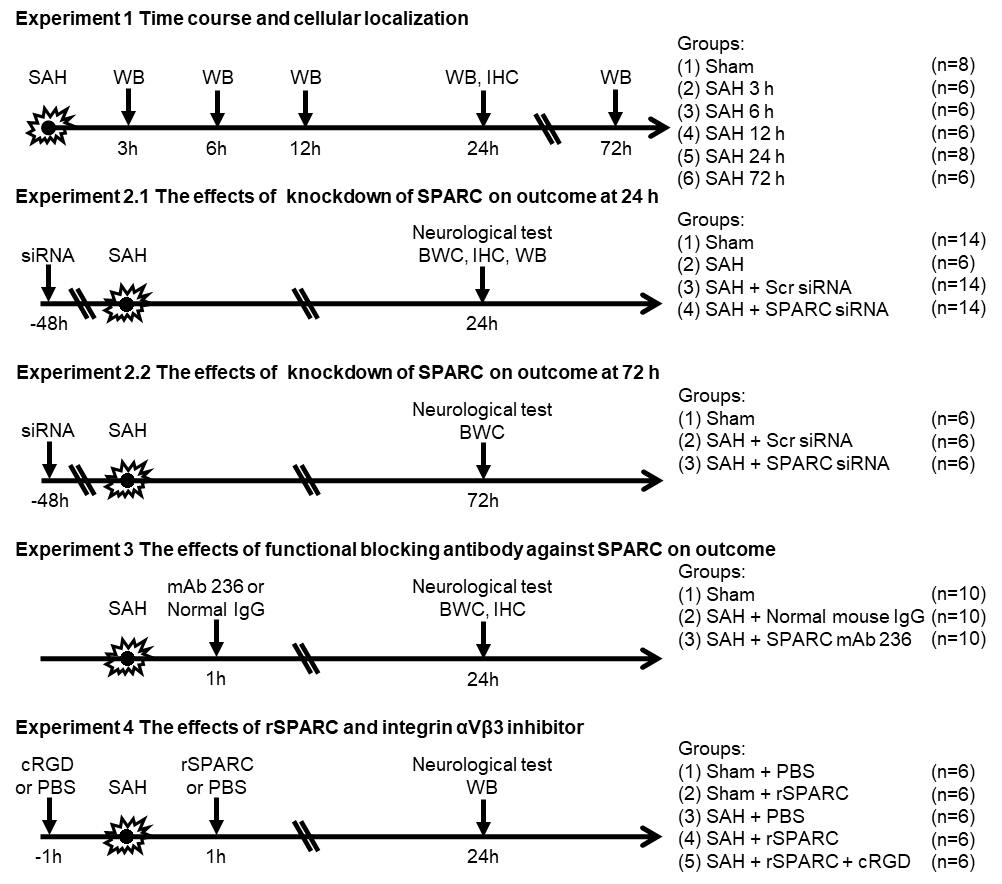
**

**Figure S1 Experimental design and animal groups.** BWC, brain water content; cRGD, cyclo(-RGDfK): integrin αVβ3 inhibitor; IgG, immunoglobulin G; IHC, immunohistochemistry; mAb, monoclonal antibody; PBS, phosphate buffered saline; SPARC, secreted protein acidic and rich in cysteine; rSPARC, recombinant SPARC; SAH, subarachnoid hemorrhage; siRNA, small interfering ribonucleic acid; Scr siRNA, scrambled siRNA; WB, Western blot.

**a**

| Test | Score | | | |
| --- | --- | --- | --- | --- |
|  | **0** | **1** | **2** | **3** |
| Spontaneous Activity  (in cage for 5 min) | No movement | Barely moves position | Moves but does not approach at least 3 sides of cage | Moves and approaches at least 3 sides of cage |
| Spontaneous movements of all limbs | No movement | Slight movement of limbs | Moves all limbs  but slowly | Moves all limbs same as pre-SAH |
| Movements of forelimbs (outstretching while held by tail) | No outstretching | Slight outstretching | Outstretching limited and less than pre-SAH | Outstretching same as pre-SAH |
| Climbing wall  of wire cage | --------- | Fails to climb | Climbs weakly | Normal climbing |
| Response to both side of trunk touch | --------- | No response | Weak response | Normal response |
| Response to vibrissae touch | --------- | No response | Weak response | Normal response |

| Test | Score | | | | |
| --- | --- | --- | --- | --- | --- |
|  | **0** | **1** | **2** | **3** | **4** |
| Beam walking  (60 sec) | No walking and falls off | No walking but remains on beam | Walking but falls off | Walking less than 20 cm | Walking beyond 20 cm |

**b**

**Figure S2 Neurobehavioral tests. a** Modified Garcia’s score. **b** Beam balance test.

**
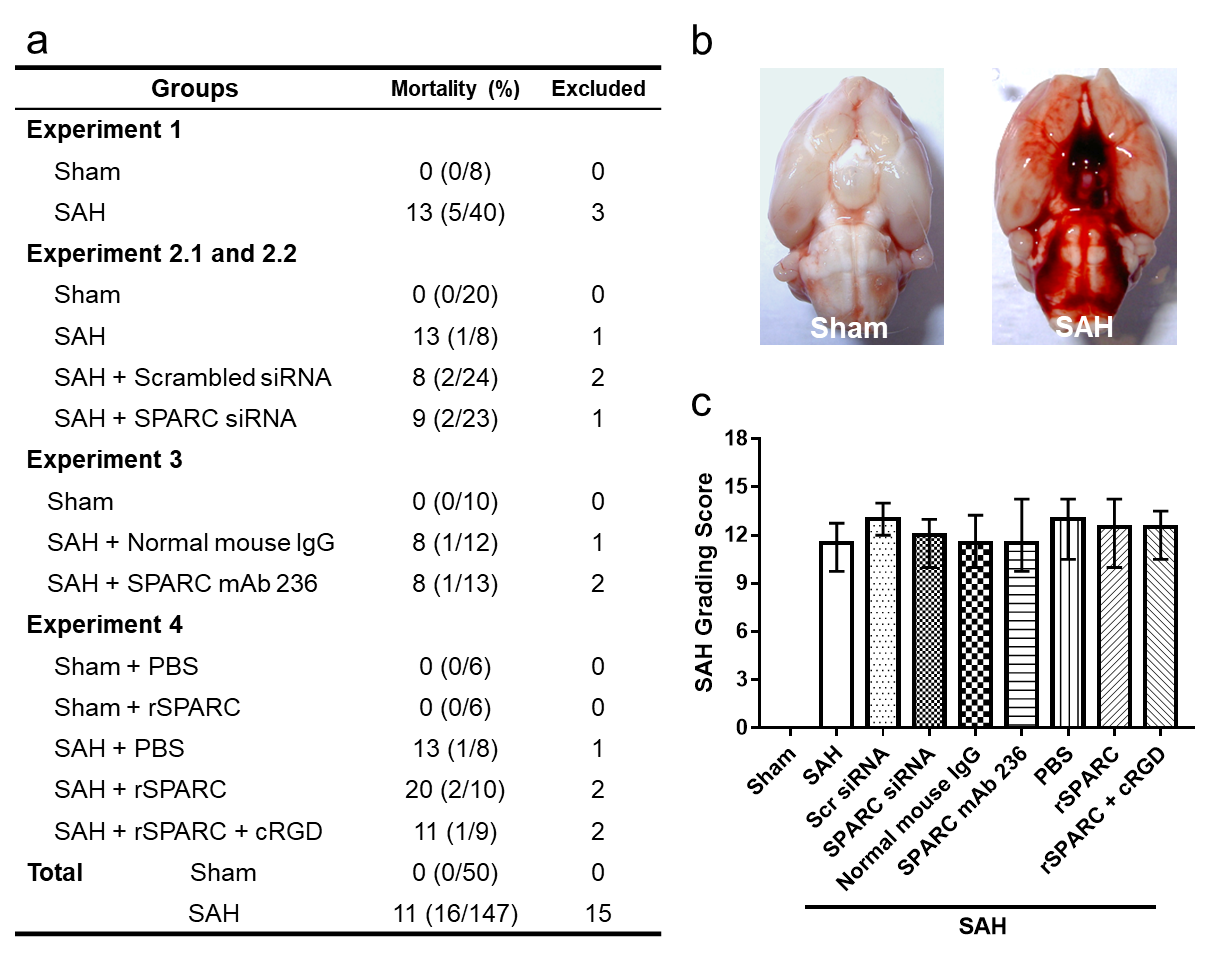
**

**Figure S3** **Mortality and subarachnoid hemorrhage (SAH) grade.** **a** Animal usage and mortality of all experiment groups. **b** Representative basal image of brain in animal models. **c** SAH grade scores of all groups. Data are expressed as median ± 25th-75th percentiles. cRGD, cyclo(-RGDfK): integrin αVβ3 inhibitor; IgG, immunoglobulin G; mAb, monoclonal antibody; PBS, phosphate buffered saline; siRNA, small interfering ribonucleic acid; Scr siRNA, scrambled siRNA; SPARC, secreted protein acidic and rich in cysteine; rSPARC, recombinant SPARC.


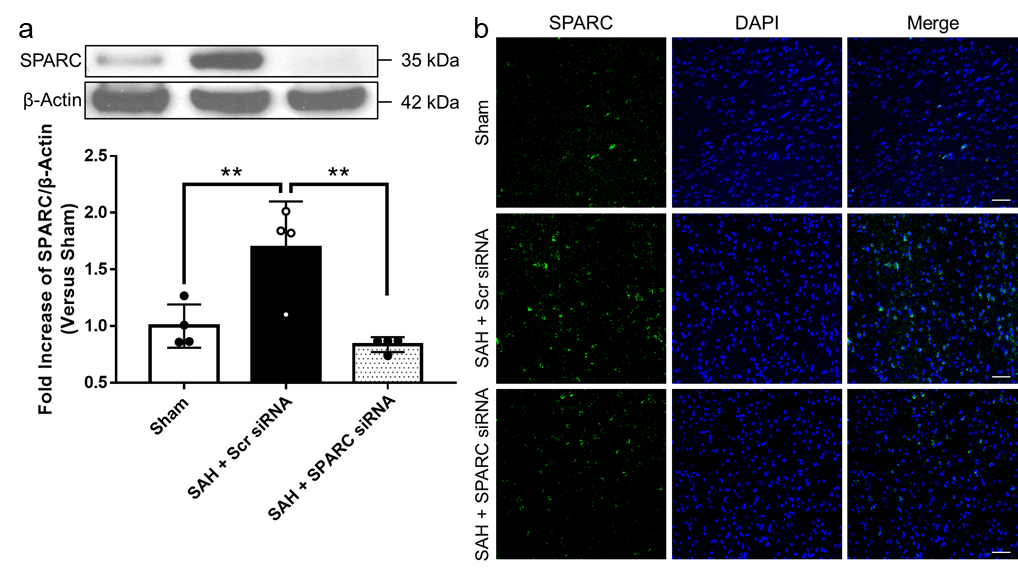


**Figure S4 The knockdown efficiency of a secreted protein acidic and rich in cysteine (SPARC) small interfering ribonucleic acid (siRNA) at 24 h after subarachnoid hemorrhage (SAH) induction. a** Representative Western blot images and densitometric quantification of expressions of SPARC. Expression levels of SPARC are divided by the levels of β-actin and expressed as a ratio of the average levels of Sham models for normalization (mean ± standard deviation; n = 4 per group). ** *P* < 0.01. **b** Representative immunohistochemical staining of SPARC in the left temporal cortex at 1.0 mm posterior to the bregma. Nuclei are stained with DAPI (blue). Scale bar = 50 μm (n = 4 per group).
